# *Stenotrophomonas maltophilia* natural history and evolution in the airways of adults with cystic fibrosis

**DOI:** 10.3389/fmicb.2023.1205389

**Published:** 2023-06-15

**Authors:** Conrad Izydorczyk, Barbara J. Waddell, Christina S. Thornton, John M. Conly, Harvey R. Rabin, Ranjani Somayaji, Michael G. Surette, Deirdre L. Church, Michael D. Parkins

**Affiliations:** ^1^Department of Microbiology, Immunology, and Infectious Diseases, Cumming School of Medicine, University of Calgary, Calgary, AB, Canada; ^2^Department of Medicine, Cumming School of Medicine, University of Calgary and Alberta Health Services, Calgary, AB, Canada; ^3^Cumming School of Medicine, Snyder Institute for Chronic Diseases, University of Calgary and Alberta Health Services, Calgary, AB, Canada; ^4^Department of Pathology and Laboratory Medicine, Cumming School of Medicine, University of Calgary and Alberta Health Services, Calgary, AB, Canada; ^5^Department of Biochemistry and Biomedical Sciences, McMaster University, Hamilton, ON, Canada

**Keywords:** cystic fibrosis, bronchiectasis, *Stenotrophomonas maltophilia*, infection transmission, genomics, epidemiology, evolution, natural history

## Abstract

**Introduction:**

*Stenotrophomonas maltophilia* is an opportunistic pathogen infecting persons with cystic fibrosis (pwCF) and portends a worse prognosis. Studies of *S. maltophilia* infection dynamics have been limited by cohort size and follow-up. We investigated the natural history, transmission potential, and evolution of *S. maltophilia* in a large Canadian cohort of 321 pwCF over a 37-year period.

**Methods:**

One-hundred sixty-two isolates from 74 pwCF (23%) were typed by pulsed-field gel electrophoresis, and shared pulsotypes underwent whole-genome sequencing.

**Results:**

*S. maltophilia* was recovered at least once in 82 pwCF (25.5%). Sixty-four pwCF were infected by unique pulsotypes, but shared pulsotypes were observed between 10 pwCF. In chronic carriage, longer time periods between positive sputum cultures increased the likelihood that subsequent isolates were unrelated. Isolates from individual pwCF were largely clonal, with differences in gene content being the primary source of genetic diversity objectified by gene content differences. Disproportionate progression of CF lung disease was not observed amongst those infected with multiple strains over time (versus a single) or amongst those with shared clones (versus strains only infecting one patient). We did not observe evidence of patient-to-patient transmission despite relatedness between isolates. Twenty-four genes with ≥ 2 mutations accumulated over time were identified across 42 sequenced isolates from all 11 pwCF with ≥ 2 sequenced isolates, suggesting a potential role for these genes in adaptation of *S. maltophilia* to the CF lung.

**Discussion:**

Genomic analyses suggested common, indirect sources as the origins of *S. maltophilia* infections in the clinic population. The information derived from a genomics-based understanding of the natural history of *S. maltophilia* infection within CF provides unique insight into its potential for in-host evolution.

## 1. Introduction

*Stenotrophomonas maltophilia* is an opportunistic gram-negative pathogen increasingly recognized for its potential to cause a variety of human infections (Brooke, 2021), particularly among immunocompromised individuals such as those with cystic fibrosis (CF). The overall prevalence of *S. maltophilia* in persons with CF (pwCF) has increased in recent decades (Salsgiver et al., 2016; Hatziagorou et al., 2020) and has remained relatively steady in Canada in recent years (∼15%) (Cystic Fibrosis Canada, 2021). Chronic lung infections with *S. maltophilia* have been associated with adverse clinical outcomes, including increased pulmonary exacerbation frequency, hospitalization, requirements for intravenous antibiotic treatments (Waters et al., 2011; Berdah et al., 2018), poorer baseline health (Com et al., 2014; Berdah et al., 2018), variably accelerated lung function decline (Waters et al., 2012; Cogen et al., 2015; Barsky et al., 2017; Berdah et al., 2018), and a higher risk of progression to end-stage lung disease (Waters et al., 2013). However, the role of *S. maltophilia* in CF is not fully resolved, as some studies have not found any associations between infection and adverse clinical outcomes (Karpati et al., 1994; Goss et al., 2002, 2004; Marchac et al., 2004).

Several studies have investigated the natural history of *S. maltophilia* infection in CF (Vidigal et al., 2014; Pompilio et al., 2016, 2020; Barsky et al., 2017; Chung et al., 2017; Esposito et al., 2017). In general, *S. maltophilia* infection appears to be short-lived in many individuals (Krzewinski et al., 2001). However, in those with repeated, persistent isolation of *S. maltophilia* from sputum cultures over time, infection by multiple genotypes over time is common (Vidigal et al., 2014; Pompilio et al., 2016; Chung et al., 2017; Esposito et al., 2017). In contrast, co-infection by multiple distinct, non-clonal sub-strains and cross-infection of multiple patients by the same strain are infrequent (Demko et al., 1998; Denton et al., 1998; Krzewinski et al., 2001; Vidigal et al., 2014). Moreover, an initial infecting *S. maltophilia* strain may adapt to the CF lung environment, diversifying under selective pressures into clonally-derived sub-lineages (Chung et al., 2017). In some studies, hypermutation has also been reported in approximately 30% of *S. maltophilia* strains recovered from pwCF (Vidigal et al., 2014; Esposito et al., 2017).

Studies of *S. maltophilia* natural history in CF are limited, however, in their inclusion of relatively small numbers of pwCF, with a focus on those with chronic infection, short duration of follow-up, and use of low-resolution molecular typing methods (Vidigal et al., 2014; Pompilio et al., 2016, 2020); only a single study used whole-genome sequencing for strain assessment in multiple pwCF over time (Esposito et al., 2017). Further, none of these studies investigated the potential for *S. maltophilia* to spread between pwCF.

Herein, we performed a retrospective investigation of the natural history and potential for clinic-associated, patient-to-patient transmission of *S. maltophilia* at a greater resolution and across a large cohort. We drew on the Calgary Adult CF Biobank, which includes every isolate from every clinical encounter from the entire CF cohort attending the clinic. The objectives of this study were to (i) assess the patterns of infecting *S. maltophilia* isolates and strains, (ii) to determine whether infection transmission may have been a source of new *S. maltophilia* infections at our clinic, and (iii) to identify any associations between infecting strain patterns and clinical outcomes.

## 2. Materials and methods

### 2.1. Patient population and strains

In this retrospective single-center cohort study, we analyzed *S. maltophilia* isolates from pwCF attending the Southern Alberta Adult CF Clinic between 1979 and 2016. Clinical practice directs that all pwCF should receive routine quarterly sputum testing and as required clinically (e.g., during exacerbations). Each pathogen recovered from real-time clinical investigations is frozen at −80°C and included in the Southern Alberta Adult CF Clinic Biobank. Each distinct colony morphotype of each pathogen is collected and frozen, separately.

Inclusion criteria for pwCF in this study included a confirmed diagnosis of CF (Farrell et al., 2008), aged ≥ 18 years, and ≥ 1 *S. maltophilia* positive sputum cultures collected. PwCF entering the cohort who had received a life-saving lung transplant were excluded, and those receiving transplant during follow-up were censured at the time of transplant. This study received approval from the University of Calgary’s Conjoint Health Research Ethics Board (REB-15-2744).

### 2.2. Bacterial strain typing

To assess for strain diversity and relatedness given the magnitude of samples in the CACFC Biobank, representative yearly *S. maltophilia* isolates from all pwCF with ≥ 1 *S. maltophilia* positive sputum cultures were typed by pulsed-field gel electrophoresis (PFGE) using protocols adapted from Parkins et al. (2014). Representative isolates were selected according to the following criteria: (1) for pwCF with a single *S. maltophilia* positive sputum culture, isolates from that time point were assessed; (2) for pwCF with ≥ 2 *S. maltophilia* positive sputum cultures, the very first and last isolates, as well as one isolate per year for each intermediate year with a *S. maltophilia* positive sputum culture, were selected. If the very first isolate was not viable, then the next earliest isolate was selected; if no viable isolate was available for a given year, then that year was skipped. In those rare situations where more than one *S. maltophilia* morphotype was identified in a single sputum sample, all isolates were assessed. Pulsotypes differing by ≤ 3 bands with ≥ 80% similarity were considered to potentially represent the same strain (Tenover et al., 1995). Shared pulsotypes were defined as those representing the same strain and found in ≥ 2 pwCF.

Two groups of isolates were selected for whole genome sequencing (WGS): (i) isolates belonging to all shared strains and (ii) isolates belonging to a selected number of non-shared strains (i.e., present in only a single patient). The former was sequenced to assess for potential transmission between patients; the latter were selected as a comparison set to allow for the observation of intra-patient genetic distances in the absence of infection transmission. In total, 34 isolates belonging to shared pulsotypes, 17 isolates from five non-shared pulsotypes, and three isolates initially identified as belonging to shared pulsotypes were sequenced. Genomic DNA was extracted using the Promega Wizard^®^ Genomic DNA Purification Kit. Genomic libraries were prepared using the Nextera XT DNA Library Prep Kit and sequenced using either an Illumina HiSeq (2 bp × 250 bp reads) or MiSeq (2 bp × 300 bp reads) instrument.

### 2.3. Public genomes (lineages analysis)

Publicly available *S. maltophilia* genomes were used to supplement those of our clinic cohort to better understand the placement of our genomes amongst the *S. maltophilia* species complex (Supplementary Table 1). Ten genomes from each of the 23 *S. maltophilia* lineages identified by Gröschel et al. (2020) were downloaded and processed.

### 2.4. Bioinformatic analyses

The full details of bioinformatic analyses are described in the Supplementary methods. In brief, sequencing reads were trimmed using Trimmomatic (Bolger et al., 2014) (v0.39) and *in silico* multi-locus sequence typing (MLST) performed with stringMLST (Gupta et al., 2017) (v0.6.3). Isolate genomes were assembled with Unicycler (Wick et al., 2017) (v0.4.8) and annotated using RASTtk as implemented in the PATRIC Command Line Interface toolkit (Davis et al., 2020) (v1.035). Pangenome analysis was performed using Panaroo (Tonkin-Hill et al., 2020) (v1.2.8). Core genome phylogenies were generated using IQ-Tree (Minh et al., 2020), and gene presence/absence clustering was performed using the Ape (Paradis et al., 2004) (v5.3) R package.

Single-nucleotide polymorphism (SNP) calling was performed (a) in an ST-specific manner and (b) for all isolates sequenced in this work against a single reference (*S. maltophilia* strain K279a, GCF_000072485.1) using Snippy (Seemann, n.d.a) (v4.6.0). Transition/transversion rates were calculated using VCFtools (Danecek et al., 2011) (v0.1.16) to identify putative hypermutating strains. Reference genomes for ST-specific SNP calling are found in Supplementary Table 2. Phylogenies were generated using IQ-Tree (Minh et al., 2020) (v2.0.3) and corrected for recombination using ClonalFrameML (Didelot and Wilson, 2015) (v1.12). Snp-dists (Seemann, n.d.b) (v0.7.0) was used to obtain pairwise SNP distance matrices.

Mutation rates for individual STs were calculated using TempEst (Rambaut et al., 2016) (v1.5.3), while the overall SNP accumulation rate was calculated using the lme4 R package (Bates et al., 2015) by fitting a linear mixed effects model to pairwise SNP distances, where the dependent variable was the SNP distance, independent variable the time between isolate collection dates, and patient IDs were included as a mixed effect (Coll et al., 2020).

### 2.5. Transmission analysis

The potential for transmission to have occurred between pwCF infected with the same *S. maltophilia* STs was simultaneously assessed with four complementary analyses, each offering a different type of support for a hypothesis of transmission. These analyses included: (i) SNP/wgMLST allele distance support: inter-pwCF isolate pairs with SNP/wgMLST allele distances overlapping with the distribution of intra-pwCF distances; this latter distribution was compiled in an ST-specific manner but then combined across all sequenced (shared and non-shared) STs, (ii) phylogenetic support: mixed clustering/interspersal of isolates from ≥ 2 pwCF within the same clade with strong UltraFast bootstrap support (≥ 95%), (iii) gene content support: mixed clustering/interspersal of isolates from ≥ 2 pwCF within the same clade based on neighbor joining clustering, and (iv) concurrent carriage support: detection of ≥ 1 *S. maltophilia* positive sputum cultures within 6 months in a given patient pair. The combination of carriage support and at least two other analyses would warrant an individual case review examining evidence that involved patients attended clinic/hospital or other healthcare encounter within 48 h of each other. The effect of cumulative support from all four analyses would be required to support a hypothesis of transmission between a pair of patients. A lack of support in any analysis was considered to exclude the possibility of transmission.

### 2.6. Multi-mutated genes analysis

Complete details of how multi-mutated genes were identified are presented in the Supplementary methods. In brief, for each ST, we identified all pwCF with ≥ 2 sequenced isolates; this included 42 isolates from 11 pwCF across nine STs (STs 5, 23, 91, 199, 220, 224, 246, 365, and Novel 2). To identify genes that accumulated mutations during infection in the CF lung (termed SmCF genes), we filtered mutations on a person-by-person basis, retaining only those mutations that segregated within a given pwCF’s isolates regardless of whether they also segregated between isolates from different pwCF. We did not differentiate whether mutations occurred relative to a pwCF’s earliest sequenced isolate, but simply noted whether a gene had any mutations present. Assuming a clonal bacterial population within a pwCF, mutations segregating between clonal isolates collected over time would represent putatively adaptive mutations. In contrast, to identify genes with mutations accumulated outside of infection in the CF lung (termed non-adaptive genes), within each ST, we identified all mutations segregating between isolates of different pwCF but not among isolates from any pwCF. Assuming no infection transmission, such mutations would represent those defining separate strains of *S. maltophilia* and would have arisen prior to infection in the CF lung.

Each SmCF and non-adaptive gene was then classified as multi-mutated if it had ≥ 2 mutations, and multi-mutated genes were further subdivided into across-ST or within-ST, depending on which STs the contributing mutations occurred in. The distributions of synonymous, non-synonymous, and stop-codon introducing mutations were then compared between multi-mutated and non-multi-mutated genes using Fisher’s exact tests in GraphPad Prism (v9.4.1).

SmCF genes were also analyzed for enrichment of GO categories using OmicsBox (v2.1.14). Gene sequences were obtained from annotated isolate assemblies and Blast run via CloudBlast as implemented in OmicsBox. Association testing was performed with Fisher’s exact tests via the Enrichment Analysis tool, with correction for multiple testing performed by the Benjamini-Hochberg procedure and the false discovery rate set to 0.05.

### 2.7. Statistical analyses

Characteristics of the pwCF cohort were descriptively summarized. Associations between clinical/demographic factors and patients with included/excluded isolates, and time between PFGE-typed isolates from *S. maltophilia* positive sputum cultures and detection of new/any prior pulsotypes previously identified in a pwCF, were performed using Fisher’s exact tests in SPSS (v28.0.1.0). Statistical analyses for associations between clinical outcomes and carriage of multiple/shared strains were performed using longitudinally collected clinical data in R v.4.1.1 (R Core Team, 2021). All *P*-values were adjusted for multiple comparisons using the Holm-Bonferroni method (*T*-test or ANOVA). Categorical variables were presented as numbers and frequencies. Continuous variables were presented as mean ± standard deviation (SD) or median (interquartile range), as appropriate. End-stage lung disease was defined as percent predicted forced expiratory volume in 1 s (ppFEV_1_) as less than or equal to 40.

## 3. Results

### 3.1. Study and sample population

**FIGURE 1:**
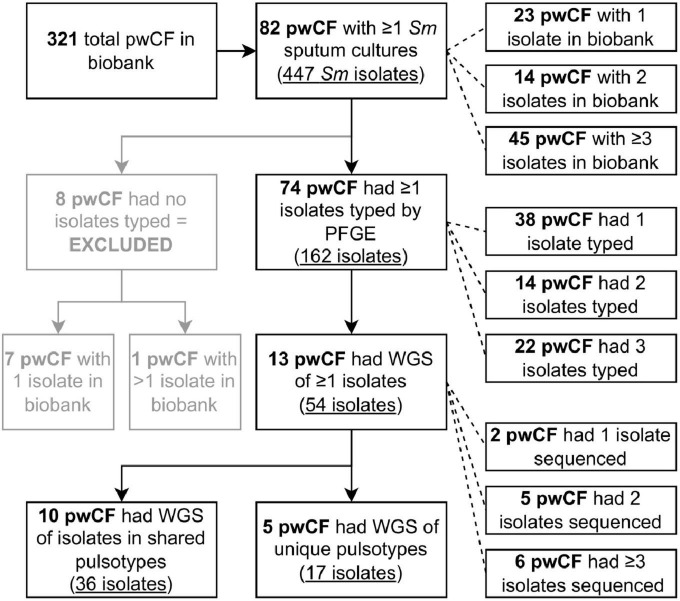
Flowchart detailing the number of pwCF and isolates identified and used at different stages of the study. Two of the pwCF included in the five reported with unique pulsotypes sequenced also had isolates in shared pulsotypes and so are double counted. *Sm*, *Stenotrophomonas maltophilia*.

Between 1979 and 2016, 321 individuals with CF were followed by the Southern Alberta CF Clinic, representing 2,640.64 person-years of observation. A flowchart of pwCF and isolate numbers used throughout the study is presented in Figure 1. Over the course of the study, 424 sputum cultures positive for *S. maltophilia* and 447 unique *S. maltophilia* morphotypes/isolates (median 1, mean 1.05 isolates/culture, range 1–2) were stored within the clinic biobank. These isolates were collected from 82/321 (25.5%) pwCF, who were followed for a median of 10.1 years (IQR 6.2–17.6 years). Twenty-three (28%) of these 82 pwCF had only one isolate in the biobank, 14 (17.1%) had two, and 45 (54.9%) had ≥ 3; the median number of isolates in the biobank per pwCF was three (IQR 1–5, range 1–65). The average prevalence of pwCF with ≥ 1 *S. maltophilia* positive sputum cultures at the clinic in any given 5-year (extended window of greater length than most persistent infections) and 1-year (short-term) window during the study period was 16.2% (IQR 12.9–17.6%) and 8.74% (IQR 4.48–10.3%), respectively. Characteristics of the 82 pwCF with at least one *S. maltophilia* positive sputum culture during the study period are presented in Table 1, and the natural history of their isolates is displayed in Supplementary Figure 1.

**TABLE 1:** Summary characteristics of pwCF with at least one *S. maltophilia* positive sputum culture between 1979 and 2016.

**Demographics^1^**	
Age at first episode (median, IQR) (years)	24.9 (20.3–32.3)
Sex (% female) (n/N)	58.5 (48/82)
**CF characteristics and comorbidities^1^**	
F508del homozygous (%) (n/N)	66.2 (45/68)
F508del heterozygous (%) (n/N)	23.5 (16/68)
Pancreatic insufficient (%) (n/N)	90.0 (72/80)
CFRD (%) (n/N)	22.5 (18/80)
CFLD (%) (n/N)	18.8 (15/80)
Osteopenia (%) (n/N)	43.0 (34/79)
Osteoporosis (%) (n/N)	7.59 (6/79)
**Determinants of disease state^1^**	
ppFEV1 (median, IQR)	59 (38–76.5)
% with mild CF lung disease (ppFEV1 predicted ≥ 70) (n/N)	32.4 (24/74)
% with moderate CF lung disease (40 < ppFEV1 < 70) (n/N)	40.5 (30/74)
% with severe CF lung disease (ppFEV1 ≤ 40) (n/N)	27.0 (20/74)
ppFVC (median, IQR)	81.5 (58–93.3)
Use of supplemental oxygen (%) (n/N)	22.5 (18/80)
Use of enteral nutrition (%) (n/N)	12.5 (10/80)
**Co-infections^1^**	
% with PA co-infection (n/N)	63.51 (47/74)
% with MSSA co-infection (n/N)	44.6 (33/74)
% with MRSA co-infection (n/N)	4.05 (3/74)
% with HI co-infection (n/N)	6.76 (5/74)
**Disease modifying CF medications^1^**	
Inhaled tobramycin (%) (n/N)	30.0 (24/80)
Inhaled colistin (%) (n/N)	1.25 (1/80)
Inhaled aztreonam (%) (n/N)	2.50 (2/80)
Azithromycin (%) (n/N)	12.5 (10/80)
Inhaled DNase (%) (n/N)	50.0 (40/80)
Inhaled hypertonic saline (%) (n/N)	15.0 (12/80)

^1^All values were based on demographic and clinical factors at first *S. maltophilia* infection episode (first positive sputum culture). F508del, deletion of phenylalanine at position 508 in protein polypeptide; CFRD, cystic fibrosis related diabetes; CFLD, cystic fibrosis liver disease; ppFEV1, percent predicted forced expiratory volume in 1 s; ppFVC, percent predicted forced vital capacity; PA, *Pseudomonas aeruginosa*; MSSA, methicillin susceptible *Staphylococcus aureus*; MRSA, methicillin resistant *Staphylococcus aureus*; HI, *Haemophilus influenzae*.

A total of 162 isolates were typed by PFGE from 74/82 pwCF (90.2%), with a median of 1 isolate typed per pwCF [interquartile range (IQR) 1–3, maximum 15] (Figure 1 and Supplementary Figure 2), spanning a collective 397.5 person-years of observation. Isolates from the remaining eight pwCF were either not recoverable by culture or missing from the biobank. These pwCF did not differ by age, sex, dF508 homozygosity, pancreatic insufficiency status, or ppFEV1 at incident isolate(s) from those with typed isolates but were more likely to have only one *S. maltophilia* positive sputum culture (7/8 vs. 16/74 patients with one isolate, Fisher’s exact test *p* = 0.0004).

### 3.2. Bacterial strain typing

A variety of pulsotypes were identified amongst recovered isolates (Supplementary Figures 1, 3). Most pwCF (54/74, 73.0%) were infected by a single pulsotype (38 pwCF had only one isolate typed), but infection over time by different pulsotypes was also observed. Across all pwCF, the median number of recovered pulsotypes was one (IQR 1–2), but this increased to two (IQR 1–2) among pwCF with ≥ 2 typed isolates (Supplementary Table 3): 15 (20.3%), three (4.1%), and two (2.7%) pwCF had infection by two, three, or four pulsotypes, respectively. Individual pulsotypes were recovered from one or a few sputum cultures and were transient or quickly replaced, and sputum cultures negative for *S. maltophilia* frequently separated cultures with different pulsotypes. The average duration of persistence of pulsotypes recovered from ≥ 2 sputum cultures was 583.1 days (median 448 days, IQR 238–861). Most pwCF (64/74, 86.5%) were infected by pulsotypes unique to themselves, but five pulsotypes encompassing 36 isolates from 10 pwCF were shared among ≥ 2 pwCF (Table 2).

**TABLE 2:** Shared *S. maltophilia* sequence types identified among patients attending the Calgary Adult CF Clinic.

Sequence type	Corresponding pulsotype(s)	Number of isolates^2^	Number of patients^3^	Patients
**Shared pulsotypes/sequence types**				
5	A	7	5	A057, A061, A275, A372, A376
39	B	2	2	A061, A200
199	C, F^1^	19	3	A055, A057, A090
220	D	3	2	A090, A371
224	E	3	2	A013, A200
**Non-shared (control) pulsotypes/sequence types^4^**				
23	G	4	1	A130
91	H	5	1	A374
246	I	2	1	A057
365	J	3	1	A344
Novel 2	K	3	1	A090

^1^A single isolate (SM003) belonging to patient A055 belonged to a unique pulsotype (F) but was included due to sharing an ST with pulsotype C isolates. All pulsotype C isolates were more closely related to each other than to the pulsotype F isolate.

^2^Total number of isolates is 54; 3 isolates that did not correspond to any shared/non-shared STs are not included here.

^3^Sum of number of patients is greater than total number of patients with shared pulsotypes due to some patients having isolates belonging to ≥ 2 shared pulsotypes.

^4^Selected non-shared sequence types were additionally included as controls for intra-patient genetic diversity.

Recovery of a new pulsotype after detection of a prior pulsotype was significantly associated with time between typed cultures (Table 3). When considering intervals of greater vs. less than one, two, and five years, the probability and odds of detection of a new pulsotype relative to a prior pulsotype increased with recovery time between typed isolates, and the relative risk of detection of a new pulsotype was greater in longer than shorter intervals. Recovery of a prior pulsotype after detection of another was rare and observed in only four (5.4%) pwCF (Supplementary Figure 1 pwCF A057, A090, A145, and A357). In all cases, re-recovery of the prior pulsotype occurred < 1 year after detection of the new pulsotype.

**TABLE 3:** Odds and relative risks of increasing time between typed *S. maltophilia* sputum cultures and likelihood of detection of a new vs. prior pulsotype.

Time between typed cultures (X years)	Probability of new pulsotype (%)	Probability of prior pulsotype (%)	Odds new vs. prior pulsotype	Probability of new pulsotype in ≤ X years (%)	Relative risk (new pulsotype > X/ ≤ X) (95% CI)	Unadjusted *P*^1^
> 1	21/37 (56.8)	16/37 (43.2)	1.31	11/50 (22.0)	2.58 (1.43–4.67)	0.00147
> 2	15/18 (83.3)	3/18 (16.7)	5.00	17/69 (24.6)	3.38 (2.13–5.37)	0.00008
> 5	9/11 (90.9)	1/11 (9.1)	9.00	22/76 (28.9)	3.14 (2.11–4.68)	0.000131

^1^Raw p-values not corrected for multiple testing. Columns presenting the probability (risk) of a new or prior pulsotype report the number of intervals between PFGE typed isolates (across all pwCF) of length > X years (first column in table) after which a new or previously detected pulsotype was detected, over the total number of intervals of each type. The “Risk of new Pulsotype in ≤ X Years” column reports the number of intervals of length ≤ X years after which a new pulsotype was detected, over the total number of intervals of length ≤ X years.

### 3.3. Genetic diversity of sequenced isolates

To examine infection dynamics at a higher resolution than PFGE allows and determine whether any instances of infection transmission may have taken place, we sequenced a total of 54 *S. maltophilia* isolates (Table 2). These included 36 isolates from 10 pwCF belonging to shared pulsotypes, 17 isolates from five pwCF with unique pulsotypes (two of these pwCF also had shared pulsotypes) to serve as non-shared controls to aid in establishment of genetic distance thresholds, and one isolate of a unique pulsotype (pulsotype F) belonging to a pwCF (A055) with 14 other isolates in pulsotype C.

**FIGURE 2:**
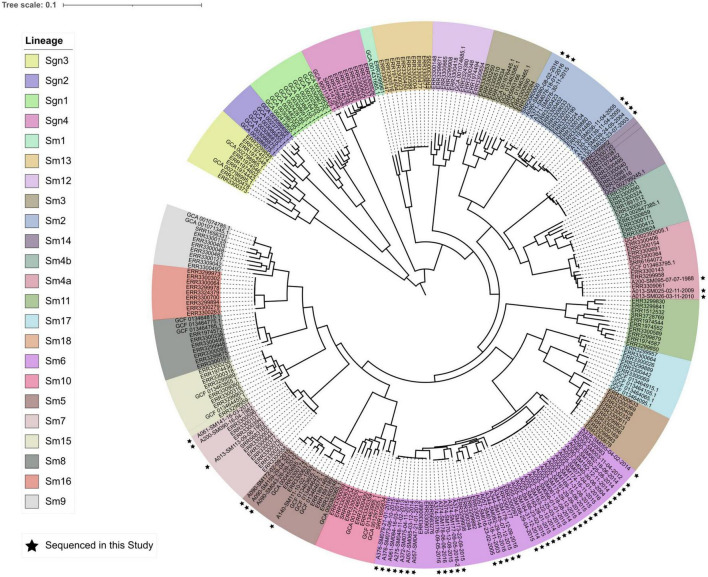
Core genome phylogeny (midpoint rooted) of 23 phylogenetic lineages comprising the *S. maltophilia* species complex. Lineage colors are presented in the same order in the legend as in the phylogeny (clockwise, starting with lineage Sgn3). Isolates sequenced in this study are marked by black stars. The phylogeny was constructed from an alignment of 1,947 core genes.

*In silico* MLST identified 13 STs among the 54 sequenced isolates (Table 2). The five shared pulsotypes corresponded to a total of eight STs, while the five unique pulsotypes corresponded to five STs. Three of the eight STs from shared pulsotypes each consisted of only one isolate: in two instances, an isolate thought to belong to a shared pulsotype had a unique novel ST. In the third case, an isolate belonging to pulsotype B belonged to a novel ST that was a single locus variant of ST-39. The single pulsotype F isolate from pwCF A055 belonged to the same ST (199) as their other isolates. Sequenced STs and isolates appeared to be a random sample from the global pool of *S. maltophilia* diversity and were scattered among the 23 previously identified monophyletic lineages of the *S. maltophilia* species complex, although most STs belonging to the Sm6 lineage (Figure 2). Within their respective lineages, isolates clustered by ST and multiple STs were observed to comprise some lineages.

### 3.4. Distance thresholds of *S. maltophilia* lineages, STs, and strains

The number of SNPs separating STs ranged from 10^4^–10^5^, dependent on intra- vs. inter-lineage comparisons (Supplementary Table 4). In contrast, the number of SNPs separating intra-ST isolates was up to three orders of magnitude lower (10^2^–10^3^ SNPs) (Supplementary Table 5). A similar trend was observed with wgMLST allele distances, with inter-ST distances (on the order of 10^3^ alleles) being an order of magnitude greater than intra-ST distances (Supplementary Tables 6, 7).

**FIGURE 3:**
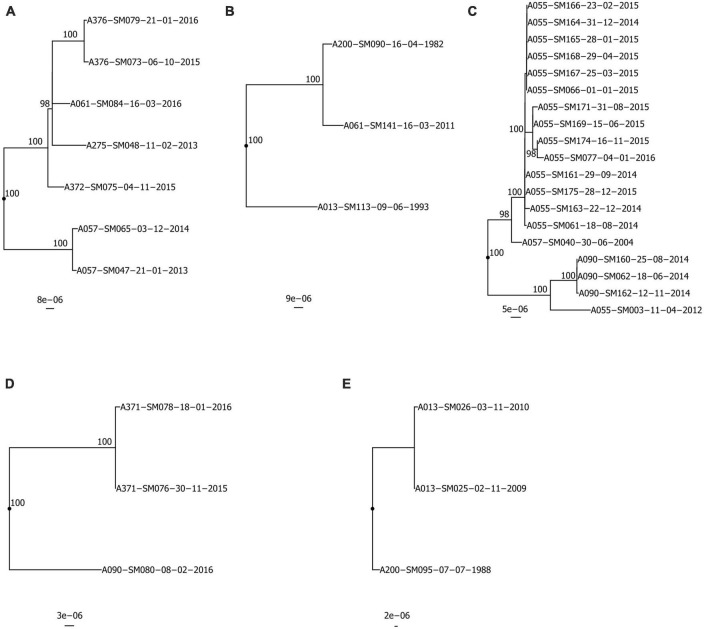
Recombination-corrected maximum likelihood phylogenies of isolates belonging to shared STs: **(A)** ST-5, **(B)** ST-39, **(C)** ST-199, **(D)** ST-220, **(E)** ST-224. Each phylogeny is rooted at the midpoint of the branch where outgroups attach. Isolate A013-SM113-09-06-1993 in **(B)** is a novel single-locus variant of ST-39 and is included as an outgroup. UltraFast bootstrap support is indicated only in clades with ≥ 95% support. Scale bars are in units of SNPs/site. Isolate names are presented in the format “Patient_Identification_Number-Isolate_Identification_Number-dd-mm-yyyy”.

**FIGURE 4:**
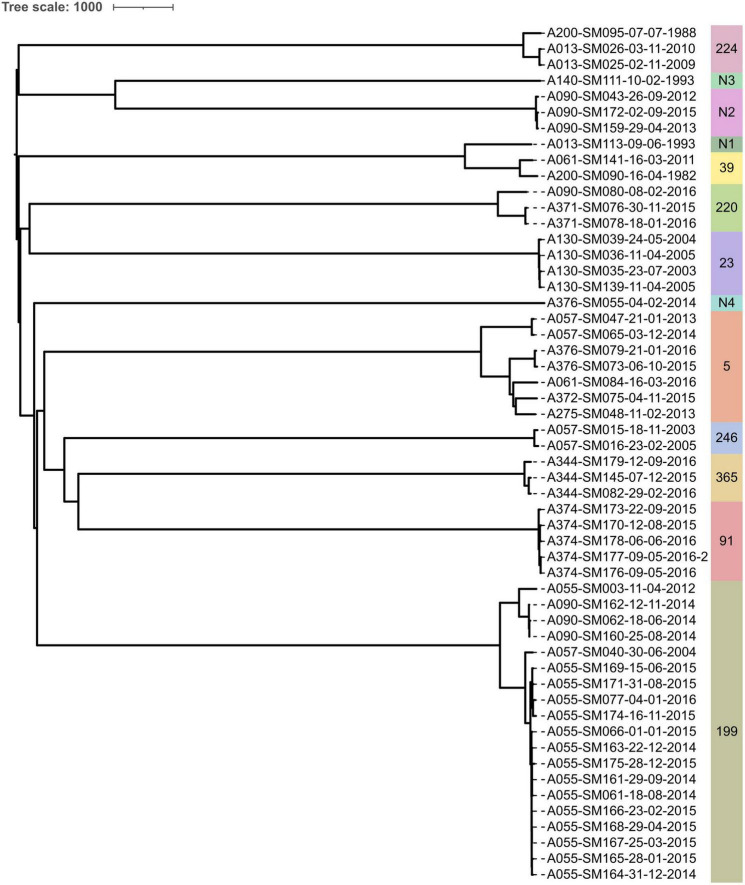
Neighbor-joining phylogeny constructed from wgMLST allele data from all isolates sequenced in this study. STs are indicated by text and colored bands on the right. Isolate names are presented in the format “Patient_Identification_Number-Isolate_Identification_Number-dd-mm-yyyy”.

Isolates from the same pwCF and of the same ST were mostly clonal in SNP and wgMLST allele phylogenies, forming pwCF-specific monophyletic clades with small genetic distances between intra-clade isolates (Figures 3, 4 and Supplementary Figure 4). Within these clades, intra-pwCF SNP distances ranged from 0 to 58 SNPs (median 10, IQR 7–22) and 2–54 wgMLST alleles (median 16, IQR 9–23) (Table 4 and Supplementary Tables 5, 7). Two exceptions to clonal relatedness were pwCF A055’s SM003 isolate (ST-199), which differed by > 200 SNPs/alleles from their other isolates and belonged to a different pulsotype, and A344’s SM137 isolate (ST-365), which was > 50 SNPs distant from their other isolates despite being collected less than 1 year apart. Excluding this latter case, the maximum intra-clade distances were 39 SNPs and 37 alleles. In contrast, intra-ST but inter-pwCF SNP and wgMLST allele distances ranged from 54 to 478 SNPs (median 253, IQR 200–258.5) and 56–461 alleles (median 260, IQR 206.5–269.5), respectively.

**TABLE 4:** Summary of intra- and inter-patient pairwise genetic distances stratified by ST.

			Intra-pwCF comparisons						Inter-pwCF comparisons		
			SNP distance		Allele distance		Gene distance		SNP distance	Allele distance	Gene distance
**Shared STs**											
ST	P1	P2	P1	P2	P1	P2	P1	P2			
5	A057	A061	18	–	17	–	15	–	430–433	426–430	1,028–1,033
	A057	A275	18	–	17	–	15	–	454–456	436–437	991–996
	A057	A372	18	–	17	–	15	–	403–407	394–395	975–980
	A057	A376	18	26	17	17	15	254	473–478	450–461	1,081–1,195
	A061	A275	–	–	–	–	–	–	193	228	364
	A061	A372	–	–	–	–	–	–	183	195	337
	A061	A376	–	26	–	17	–	254	231–240	218–229	389–583
	A275	A372	–	–	–	–	–	–	207	182	401
	A275	A376	–	26	–	17	–	254	250–254	276–293	495–695
	A372	A376	–	26	–	17	–	254	237–239	223–235	398–600
39	A061	A200	–	–	–	–	–	–	123	126	400
199	A055	A057	0–32^a^	–	2–37	–	11–457	–	54–67	56–72	318–648
	A055	A090	0–32^a^	6–10	2–37	4–7	11–457	22–263	250–269	252–271	386–828
	A057	A090	–	6–10	–	4–7	–	22–263	224–228	241–249	715–896
220	A090	A371	–	4	–	9	–	341	264–268	230–238	406–591
224	A013	A200	12	–	12	–	25	–	144–152	142–146	465–482
**Non-shared STs**											
23	A130	–	3–8	–	8–17	–	21–339	–			
91	A374	–	6–15	–	3–21	–	12–61	–			
246	A057	–	39	–	29	–	414	–			
365	A344	–	13–58	–	18–54	–	17–280	–			
N2	A090	–	7–15	–	9–22	–	6–14	–			

^a^Excluding isolate A055-SM003 (pulsotype F).

The overall rate of SNP accumulation across all patients was estimated to be 8.4 SNPs/year. Mutation rates varied by ST and ranged from 1.65 to 45.2 SNPs/year (Supplementary Table 8). Only ST-365’s mutation rate (45.2 SNPs/year) suggested a possible hypermutating strain. However, no elevated transition/transversion (Ts/Tv) ratios were observed among isolates from any ST (mean 2.14, range 1.78–2.28), including among ST-365 isolates. Further, only three isolates (ST-Novel 1 isolate SM113, ST-39 isolates SM141 and SM090) had any frameshift, loss of start codon, or stop-codon introducing mutations in genes involved in DNA mismatch repair (*mutS*, *mutL*, and *uvrD*). Specifically, these three isolates had the same 23 bp deletion in *mutL* leading to a frameshift and loss of the start codon, but none exhibited elevated mutation rates. A median of 4,472.5 coding sequences (IQR 4,311.75–4,472.5) were annotated per isolate genome. Intra-pwCF isolates exhibited greater variability in relatedness with respect to differences in gene content than SNPs/wgMLST alleles and could be as different from one another as to isolates from another pwCF. The pairwise number of genes present/absent among intra-pwCF isolates ranged from 6 to 608 genes (median 86, IQR 33.5–289), whereas the inter-pwCF equivalent was 311–1,195 genes (IQR 4,311.75–4,472.5). Clustering patterns based on gene presence/absence also recovered the clonal relationships observed in SNP/wgMLST allele phylogenies but with longer branches between even closely related isolates (Supplementary Figure 5).

### 3.5. Transmission

The potential for patient-to-patient transmission of *S. maltophilia* among patients within shared STs was simultaneously investigated using four complementary analyses: SNP/wgMLST distances, phylogenetics, gene content analysis, and six-month carriage overlap. Collectively, sixteen pairs of patients were identified among the five shared STs (median one patient pair per shared ST, range 1–10), for eleven of which ≥ 2 isolates were available for at least one pwCF in the pair (Tables 4, 5). Of the sixteen pairs of patients analyzed, nine pairs had no analyses supporting transmission, and seven pairs had one analysis supporting potential for transmission. In no pairs of patients was support for transmission provided by ≥ 2 analyses.

**TABLE 5:** Assessment for infection transmission risk within patient pairs among all identified shared STs.

ST	Patient pair	SNP/wgMLST distance support	Phylogenetic support	Gene content support	Carriage support	Epidemiological support
5	A057/A061	No	No	No	No	No
	A057/A275	No	No	No	Yes	No
	A057/A372	No	No	No	No	No
	A057/A376	No	No	No	No	No
	A061/A275	No	No	No	No	No
	A061/A372	No	No	No	Yes	No
	A061/A376	No	No	No	Yes	No
	A275/A372	No	No	No	No	No
	A275/A376	No	No	No	No	No
	A372/A376	No	No	No	Yes	No
39	A061/A200	No	–^2^	No	No	No
199	A055/A057	Partial^1^	No	No	No	No
	A055/A090	No	No	No	Yes	No
A057/A090	No	No	No	No	No
220	A090/A371	No	No	No	Yes	No
224	A013/A200	No	No	No	No	No

^1^Distance support here is among SNP but not wgMLST distances, and only among 12/14 isolate pairs between these two pwCF.

^2^Only one isolate/patient was available for this pair, so phylogenetic support was unable to be assessed.

ST-specific SNP and wgMLST allele distances were smaller among intra-pwCF isolate pairs than inter-pwCF isolate pairs in all but one case. Specifically, patient A057’s single isolate (ST-199) was similarly distant to A055’s isolates as the observed SNP distances between some of patient A344’s isolates (ST-365). However, these latter distances may represent a separate, distinctly acquired sub-strain in this pwCF or a hypermutating strain, and this overlap was not observed among wgMLST allele distances. Phylogenetic support for transmission was not observed for any isolate pairs (Figure 3). In all cases where ≥ 2 isolates were available for at least one patient in a given pair, isolates clustered by patient with shorter branches to other isolates from the same patient than to isolate(s) from other patients. Similarly, gene content support was not observed, as hierarchical clustering recovered the same clonal relationships as phylogenetic analysis, albeit with longer branches between some intra-patient isolates (Supplementary Figure 5). Carriage support was the most common and observed for six pairs of isolates, likely due to the non-stringent definition of carriage support (6-month window).

### 3.6. Multi-mutated genes

One-hundred ninety-eight protein coding genes from 42 isolates belonging to 11 pwCF across nine STs (i.e., all isolates from pwCF with ≥ 2 isolate sequenced) were identified with mutations arising during infection in CF (“SmCF genes”); 1,042 genes had mutations acquired outside of CF infection (“non-adaptive genes”). Mutations in SmCF genes were more likely to be both non-synonymous (Fisher’s exact test adjusted *p* = 0.0065) and stop-codon introducing mutations (adjusted *p* = 0.0031) than synonymous, compared to mutations in non-adaptive genes. Enrichment analysis did not identify any gene ontology (GO) categories significantly associated with SmCF vs. non-adaptive genes, however.

Fourteen SmCF genes were multi-mutated over time in pwCF across STs, two were multi-mutated across and within STs, and eight were multi-mutated only within STs; three multi-mutated intergenic regions were also identified (Supplementary Table 9). Neither multi-mutated SmCF nor non-adaptive genes were more likely to have non-synonymous or stop-codon introducing mutations than synonymous mutations compared to their non-multi-mutated counterparts, respectively (Fisher’s exact test unadjusted and adjusted *p* > 0.05). Similarly, mutations in SmCF multi-mutated genes were not more likely to be non-synonymous (unadjusted and adjusted *p* > 0.05) nor stop-codon introducing mutations compared to mutations in multi-mutated non-adaptive genes, but a trend was observed among stop-codon introducing mutations (unadjusted *p* = 0.004, adjusted *p* = 0.059). When SmCF multi-mutated genes were separated into across-ST and within-ST subcategories and compared, a trend favoring non-synonymous (unadjusted *p* = 0.004, adjusted *p* = 0.055) but not stop-codon introducing (unadjusted and adjusted *p* > 0.05) mutations was observed. Neither across-ST nor within-ST multi-mutated SmCF genes were more likely to have non-synonymous or stop-codon introducing mutations than synonymous mutations compared to non-multi-mutated SmCF genes, although trends were observed among non-synonymous mutations in the across-ST (unadjusted *p* = 0.065, adjusted *p* > 0.05) and within-ST (unadjusted *p* = 0.018, adjusted *p* > 0.05) groups, and among stop-codon introducing mutations in the across-ST group (unadjusted *p* = 0.077, adjusted *p* > 0.05). No SmCF genes were found to be significantly more likely to mutate on the basis of sex, although two genes [GDP-mannose 4,6-dehydratase (EC 4.2.1.47) and an epimerase/dehydratase protein] displayed trends (unadjusted *p* = 0.083) in being more likely to mutate in male relative female pwCF.

Multi-mutated regions included a variety of genes and intergenic regions (Supplementary Table 9). Notably, two multi-mutated intergenic regions were clustered around the same set of genes involved in iron acquisition (an outer membrane hemin receptor and hemin uptake protein HemP/HmuP), both of which were also (singly) mutated during CF lung infection. Multi-mutated genes included genes associated with efflux transporters, basic metabolism, protein transport, virulence, and hypothetical proteins. For example, the *smeT* gene (a repressor of the SmeDEF efflux transporter system) had acquired two independent non-synonymous mutations, including a Leu166Gln mutation found in nine isolates from a single patient.

### 3.7. Clinical outcomes

Fifty-four pwCF (65.9%) progressed to end-stage lung disease (defined as ppFEV_1_ < 40) during their time at the clinic. Amongst those with advanced lung disease, 23 (28.0%) required lung transplantation. In total, 39 (47.6%) died during the study period. PwCF who succumbed to end-stage lung disease or received transplants were not more likely to have been infected with multiple strain types as compared to those who had stable lung function [14 (25.9%) vs. 6 (21.4%), *p* = 0.79]. Patients who were infected with a shared clone (≥ 2 patients) were not more likely progress to end stage lung disease as compared to those with stable lung function [6 (11.1%) vs. 4 (14.3%), *p* = 0.73]. In particular, infection with ST-5, infecting 5 individuals did not portend a worse prognosis (*p* = 0.83).

## 4. Discussion

We retrospectively analyzed a large, comprehensive collection of *S. maltophilia* isolates from all pwCF attending the Southern Alberta Adult CF Clinic collected over 37 years in order to understand the natural history of infection and potential for pwCF-pwCF infection transmission. Approximately a quarter of pwCF attending the clinic had ≥ 1 *S. maltophilia* positive sputum culture over the study duration, but the prevalence over time was lower and relatively constant (16.2% in 5-year windows and 8.74% in 1-year windows). This prevalence of infection in pwCF is greater than that reported in some (Goss et al., 2004) but not other (Capaldo et al., 2020) studies. While most patients had infection with only a single strain, detection of multiple *S. maltophilia* strains over time was common, as previously reported (Vidigal et al., 2014; Pompilio et al., 2016; Chung et al., 2017; Esposito et al., 2017), but co-infection was not. The persistence of pulsotypes recovered from multiple sputum cultures and their duration observed here is consistent with similar findings by Esposito et al. (2017) but is somewhat different from the lower diversity and prolonged infection by individuals strain observed for *Pseudomonas aeruginosa* (Jelsbak et al., 2007; Fernández-Olmos et al., 2013).

Individual strains were mostly clonal, with SNP and wgMLST allele distances consistent with close relatedness. However, even clonal isolates could often be differentiated by their gene content, suggesting that the gain/loss of genes may contribute more to the genetic diversity of these strains than mutation. Most patients carried unique strains, and while shared, genetically closely related strains were observed in some pwCF, patient-to-patient associated transmission, and infection within the healthcare system, was considered unlikely due to a lack of supporting evidence. In contrast to some previous studies (Vidigal et al., 2014; Esposito et al., 2017) but consistent with Pompilio et al. (2016), based on our analysis of mutation rates, transition/transversion ratios, and analysis of mutations in MMR genes, we did not identify a significant proportion of hypermutating strains in our panel of isolates. Indeed, we identified only three isolates from one patient exhibiting an elevated collective mutation rate (but not Ts/Tv ratio and no mutations in MMR genes) consistent with hypermutation. However, as only a small subset of isolates underwent WGS, it is possible hypermutators are present among non-sequenced isolates. Our estimated overall rate of mutation accumulation (8.4 SNPs/year) is consistent with a similar estimate (8 SNPs/year) from a recent study of 552 isolates from 23 sites of the lungs of a CF patient (Chung et al., 2017) and broadly consistent with some rate estimates reported for other CF pathogens (Cramer et al., 2011; Lieberman et al., 2011; Marvig et al., 2013, 2015; Markussen et al., 2014; Silva et al., 2016; Viberg et al., 2017; Gabrielaite et al., 2021; Khademi et al., 2021). Several previous studies of *S. maltophilia* in CF have calculated rifampin mutation frequencies on a per-isolate basis and observed variably increasing/decreasing rates over time (Vidigal et al., 2014; Pompilio et al., 2016; Esposito et al., 2017) but given that our rates were estimated using computational methods and per-ST, our data are not directly comparable.

A limitation of current studies of *S. maltophilia* in CF is their inclusion of relatively small numbers of patients (typically only those chronically infected) and short study periods [with infrequent studies extending up to 10 years (Esposito et al., 2017)]. Thus, comprehensive longitudinal clinic-wide assessments of *S. maltophilia* infection in CF are lacking. Further, most studies have used traditional molecular strain typing methods [rep-PCR and pulsed-field gel electrophoresis (PFGE)] for strain assessment (Vidigal et al., 2014; Pompilio et al., 2016, 2020), with only a single study using whole-genome sequencing (WGS) on multiple chronically infected patients (Esposito et al., 2017). This latter point is particularly relevant, as many studies have identified a significant proportion of patients with shared strains (as defined by molecular methods). While shared strains as determined through molecular methods may indicate the potential for infection transmission (Stapleton et al., 2020; Gabrielaite et al., 2021), it is not sufficient to identify a transmission event (Parkins et al., 2018; Doyle et al., 2020; Izydorczyk et al., 2020, 2022). This is key, as independent acquisition of the same strain without a CF intermediary is well known to occur with other CF pathogens (Doyle et al., 2020; Izydorczyk et al., 2020; Stapleton et al., 2020), confounding our ability to understand infection transmission. To date, no studies of *S. maltophilia* in CF have investigated its potential to spread between patients.

By utilizing the Calgary Adult CF Clinic Biobank−a unique, one-of-a-kind resource−we were able to provide a broad picture of *S. maltophilia* infection dynamics, genetic diversity, and potential for clinic-associated patient-to-patient infection transmission across an entire CF clinic over a period of 37 years. While previous studies of *S. maltophilia* in CF focused on detailed analyses of many isolates from individual patients (Pompilio et al., 2016; Chung et al., 2017), utilized molecular methods as a baseline for strain typing (Vidigal et al., 2014; Pompilio et al., 2020), or focused on relatively small numbers of patients over short timeframes (Esposito et al., 2017), we demonstrated the pertinence of their findings to the entire clinic level. At the same time, we were able to achieve a finer resolution in the patterns and relationships of infecting strains compared to previous works (Esposito et al., 2017) by analyzing sequenced isolates in an ST-specific manner. Indeed, it is now well recognized that the choice of reference genome in SNP calling-based studies is critical and that single-reference analyses are inadequate (Valiente-Mullor et al., 2021).

And while others have reported infection with *S. maltophilia* portends a worsened prognosis relative to those uninfected (Waters et al., 2011, 2012, 2013; Com et al., 2014; Cogen et al., 2015; Barsky et al., 2017; Berdah et al., 2018), we did not observe differences in our cohort based on whether a strain was shared or unique to a single individual, or whether pwCF carried multiple strain types over time versus were only ever infected with a single strain type, as has been observed with other species such as *P. aeruginosa* (Parkins et al., 2018).

Recently, *S. maltophilia* has been suggested to exist as a species complex consisting of 23 “species-like lineages” (Gröschel et al., 2020). This may partially explain the high level of strain diversity and rapid changes in infecting strain type observed in this work, since a very diverse pool of potentially infectious strains exists under the same species classification. Our results are in agreement with the previous finding that detection of multiple *S. maltophilia* strains over time is common (Vidigal et al., 2014; Pompilio et al., 2016; Chung et al., 2017; Esposito et al., 2017) and extend previous studies by demonstrating that this pattern may persist for several decades. This pattern of rapid strain acquisition is also consistent with a hypothesis of independent environmental acquisition as the source of new infections in pwCF in CF cohorts with adequate infection control protocols, as has been suggested for other CF pathogens (Yan et al., 2019; Doyle et al., 2020; Stapleton et al., 2020). This is further supported by a lack of epidemiological evidence for infection transmission, and the clonal nature of intra-pwCF strains. The low proportion of pwCF with shared strains here (13.5%) is consistent with some previous studies (Esposito et al., 2017). The clonal nature of intra-pwCF strains is also in line with observations of other CF pathogens (Caballero et al., 2015). No evidence of the circulation of any epidemic strains was observed, unlike what has been commonly observed in some strains of *P. aeruginosa*, *Burkholderia cenocepacia*, and *Mycobacterium abscessus massiliense* (Ledson et al., 1998; Parkins et al., 2018).

Since most intra-pwCF isolates were clonal with limited SNP and wgMLST allele diversity but could differ from one another to the same degree as from isolates from different pwCF with respect to gene content, our data suggests that gene gain/loss may be a stronger driver of *S. maltophilia* evolution in CF. Indeed, it has been suggested that *S. maltophilia* as a species evolves primarily via recombination and gene gain/loss (Yu et al., 2016), and our data supports this to be the case in CF as well. However, our analysis of mutations arising during infection in CF found that these were enriched in non-synonymous and stop codon-introducing mutations compared to mutations separating strains prior to their introduction to the CF airways, suggesting that at least some of these genes may be under adaptive pressure (Diaz Caballero et al., 2018). While not statistically significant, similar mutational spectral trends were observed for SmCF genes with multiple mutations across STs and within STs as well. Indeed, multiple independent mutations at a given locus may be indicative of adaptive pressure on the locus (Wood et al., 2005; Arendt and Reznick, 2008; Bailey et al., 2015), which we observed in sixteen loci across STs and eight loci within STs, further suggesting that selection acting on mutations is also present within these strains.

Adaptation of bacterial pathogens to the CF lung environment is well recognized, including for *S. maltophilia*, and includes changes such as attenuation of virulence, development of antimicrobial resistance, and alteration of metabolism, nutrient acquisition, and gene regulation, among others (Lieberman et al., 2011; Winstanley et al., 2016; Menetrey et al., 2021). We identified a collective 24 genes and three intergenic regions that mutated more than once across all pwCF. Indeed, the accumulation of multiple independent mutations in a given gene may be an indicator of adaptive pressures from CF-associated infections acting on that gene, as may a higher ratio of non-synonymous to synonymous mutations (Caballero et al., 2015; Diaz Caballero et al., 2018). Among multi-mutated genes, the *smeT* gene encoding a repressor of the SmeDEF efflux transporter system was mutated two times (once in two different STs). One of these mutations was a Leu166Gln mutation previously associated with SmeDEF efflux pump overexpression (Sánchez et al., 2002). Further, two doubly mutated intergenic regions associated with iron acquisition were identified. Both efflux and iron acquisition are systems known to undergo mutation and adaptation in the CF lung environment in *P. aeruginosa* and *S. maltophilia* (Winstanley et al., 2016; Menetrey et al., 2021), and we highlight these here to corroborate our results with previous works. We recognize several limitations of this work. Firstly, as a single-center retrospective analysis, we were limited to previously sampled isolates at a single Canadian clinic, with varying numbers sampled from different patients. Thus, some patients may have had denser sampling than others based on frequency of healthcare encounters. Moreover, given the magnitude of the collection in the Calgary Adult CF Clinic Biobank, only one *S. maltophilia* isolate per morphologically distinct colony type is stored per sputum culture. As such, we were limited to a single representative isolate and unable to measure strain diversity at any single point in time within a given sputum culture. In some cases, this meant that only a single isolate was available for a given pwCF, limiting the types of phylogenetic relationships and inferences (i.e., estimation of mutation rates) that could be observed for inferring transmission. The magnitude of the collection in the Calgary Adult CF Clinic Biobank also meant that we had to select at most yearly isolates per pwCF for typing so that not every *S. maltophilia* isolate was typed by PFGE (162 isolates were typed by PFGE) and not all PFGE typed isolates were sequenced (only 54 isolates underwent WGS). Selecting isolates to sequence based on PFGE typing is also a limitation in that we were limited in initial resolution by PFGE, and as we observed, PFGE pulsotypes do not always correspond to equivalent STs. Lastly, the draft nature of genome sequencing performed also means that the gene content of sequenced isolates may not be perfectly known.

In conclusion, we have demonstrated that *S. maltophilia* infection in pwCF are a random draw from the broader *S. maltophilia* species complex diversity. Infection within individual pwCF is driven by unique strains that are likely of environmental origins, as observed with other CF pathogens. While some patients may carry genetically related strains, these do not appear to be associated with patient-to-patient transmission but more likely with independent acquisition from environmental sources. The infection process is largely clonal at the SNP level, but significant diversity is present and driven by differences in gene content within strains.

## Data availability statement

The data presented in this study are deposited in the National Center for Biotechnology Information (NCBI) Short Read Archive (SRA) under the BioProject accession number PRJNA943478.

## Ethics statement

The studies involving human participants were reviewed and approved by the Conjoint Region Health Ethics Board. The patients/participants provided their written informed consent to participate in this study.

## Author contributions

CI, BW, and CT: experimentation and method development. CT, BW, HR, MS, and MP: sample and clinical data collection. MP, JC, MS, and RS: funding. CI and RS: statistical analysis. BW and MP: project management. CI and MS: bioinformatic analysis. CI: original draft. All authors have contributed to the final manuscript and approved the submitted version.
