# Supplementary material for: Stenotrophomonas maltophilia natural history and evolution in the airways of adults with cystic fibrosis
Source: Front Microbiol. 2023 Jun 15;14:1205389. doi: 10.3389/fmicb.2023.1205389 (PMC10308010; doi:10.3389/fmicb.2023.1205389)
Supplement: Supplementary file 1 [file Data_Sheet_1.docx]

Supplementary Material

*Stenotrophomonas maltophilia* natural history and evolution in the airways of adults with cystic fibrosis

Conrad Izydorczyk, Barbara J. Waddell, Christina S. Thornton, John M. Conly, Harvey R. Rabin, Ranjani Somayaji, Michael G. Surette, Deirdre L. Church, Michael D. Parkins*

*** Correspondence:** Corresponding Author: mdparkin@ucalgary.ca

# Supplementary Methods

## PULSED-FIELD GEL ELECTROPHORESIS

Pulsed-field gel electrophoresis (PFGE) was performed according to modified protocols from Parkins et al.(Parkins et al., 2014). Viable isolates were grown from glycerol frozen stock on tryptone soy yeast extract agar overnight at 37°C. Isolate DNA was digested with 20U SpeI for 4 h at 37°C with shaking and run on a 1% SeaKem® Gold agarose gel at 6 V/cm for 20 h at 10°C. The switch time was linearly ramped up from an initial time of 5 s to a final time of 45 s, with an included angle of 120°. Gels were stained with GelRed™ (Biotium 41003), and banding patterns visualized using BioNumerics (v7.6) (Applied Maths, Belgium). Relatedness between banding patterns (pulsotypes) was quantified using the Sørensen-Dice similarity coefficient with 2% position tolerance and 1.5% optimization. Dendrograms were generated using the unweighted pair-group method with arithmetic mean (UPGMA) method. Pulsotypes differing by ≤3 bands with ≥80% similarity were classified as representing the same strain. Shared strains were defined as pulsotypes found in ≥2 patients.

## BIOINFORMATIC ANALYSES

### Public Genomes for *S. maltophilia* Lineages Phylogeny

Ten genomes were randomly selected from each of the 23 *S. maltophilia* lineages as previously defined (Gröschel et al., 2020), with some of these genomes originating from prior studies(Chauhan et al., 2013; Pak et al., 2015; Roach et al., 2015; Kanamori et al., 2016; Patil et al., 2016; Esposito et al., 2017; Lira et al., 2017; Niu et al., 2017; Ochoa-Sánchez and Vinuesa, 2017; Parks et al., 2017; Yero et al., 2020). Accession numbers for selected genomes are found in **Supplementary Table 3**. If fewer than ten genomes were available for a lineage, then all available genomes were included. Genomes were downloaded using enaDataGet(enaBrowserTools, 2022) (v1.5.3) as sequencing reads if available, or as an assembly otherwise. Downloaded sequencing reads were then processed as in “Sequencing Read Trimming” and “Pangenome Analyses” below to generate a phylogeny displaying the 23 lineages of *S. maltophilia* and where the isolates sequenced in this work fall within it.

### Sequencing Read Trimming

The quality of sequencing reads for all isolates sequenced in this study, as well as reads from public genomes, was analyzed using FastQC(FastQC, n.d.) (v0.11.8). Trimmomatic(Bolger et al., 2014) (v0.39) was used to trim reads to remove sequencing adapters (option *ILLUMINACLIP:/path/to/adapters/file:2:30:8:10:true*), extra bases (option *CROP:300* for 2x300 bp sequencing reads; adjusted as required), low quality 3’ regions (option *SLIDINGWINDOW:4:5*), and ensure a minimum length of 30-31 bp (option *MINLEN:30* for isolates sequenced in this work or *MINLEN:31* for public genomes).

### *In silico* Multi-locus Sequence Typing

Sequence typing of all sequenced isolates was performed using stringMLST(Gupta et al., 2017) (v0.6.3). The *S. maltophilia* MLST database was downloaded using the command *stringMLST.py --getMLST -P /db/directory/dbname --species ‘Stenotrophomonas maltophilia’* on March 24, 2021. Sequence types were obtained using the command *stringMLST.py --predict -k 35 -z 300 -d /path/to/isolate/fastq/files/directory/ --prefix /db/directory/dbname -o /path/to/output/file*.

### *De Novo* Assembly and Annotation

*De novo* assembly of isolates sequenced in this study, as well as public genomes downloaded as sequencing reads, was performed with Unicycler(Wick et al., 2017) (v0.4.8) using trimmed reads. Unicycler was run with default settings except for the following options: *--depth_filter 0.01, --min_fasta_len 100, and --min_polish_size 100*. Assemblies for isolates sequenced in this work were then filtered to remove contigs with a sequencing depth <25% of the average chromosomal depth using a custom Python script.

*De novo* assemblies were annotated using RASTtk as implemented in the PATRIC Command Line Interface toolkit(Davis et al., 2020) (v1.035) using the default annotation workflow, with the addition of prophage calling using PhiSpy.

### Pangenome Analyses

Pangenome analysis for a) isolates sequenced in this study, and b) isolates sequenced in this study along with public genomes were performed using Panaroo(Tonkin-Hill et al., 2020) (v1.2.8). Panaroo was run with the following options for a*): --threshold 0.98 --len_dif_percent 0.98 --clean-mode strict --core_threshold 0.95 --remove-invalid-genes --aligner mafft*. Panaroo was run with the following options for b): *--aligner mafft --core_threshold 0.999 --clean-mode moderate*. Core genome phylogenies for both a) and b) were generated using IQ-Tree(Minh et al., 2020) (v2.0.3). IQ-Tree was run with 10000 UltraFast(Hoang et al., 2018) bootstrap replicates, and the best-fitting model of nucleotide evolution was selected using the IQ-Tree ModelFinder(Kalyaanamoorthy et al., 2017). Phylogenies were visualized using iTOL(Letunic and Bork, 2021) (v5).

### Whole-genome MLST (wgMLST)

WgMLST for all isolated sequenced in this work was run using chewBBACA(Silva et al., 2018) (v2.8.5) with default settings and the *S. maltophilia* wgMLST scheme developed by Gröschel et al.(Gröschel et al., 2020). A neighbor-joining tree based on allele distances was constructed using GrapeTree(Zhou et al., 2018) (v1.5.0) run with default settings and the *--missing 0* and *--method NJ* options set.

### SNP Calling and Phylogenetic Analysis

Single-nucleotide polymorphism (SNP) calling was performed using Snippy(Seemann, n.d.) (v4.6.0) in an ST-specific manner. A draft genome from each ST cluster was used as the primary reference (**Supplementary Table 4**). Sequencing reads from all isolates in an ST were aligned against the reference using Snippy with the *--unmapped* option set. Unmapped sequencing reads for each isolate were then combined for each ST and assembled with Unicycler as in “De Novo Assembly and Genome Annotation” with the following options: *--depth_filter 0.25 --min_fasta_len 100 --min_polish_size 100*. Contigs were filtered to keep only those >1000 bp and blasted using blastn(Camacho et al., 2009) (v2.9.0) against the entire ‘nt’ database (downloaded November 2021) with the *-evalue* option set to 0.00001 and the *-max_target_seqs* option set to 10. Contigs without any hits to the genus *Stenotrophomonas* were removed. Remaining contigs were combined with the original set of reference contigs and collectively annotated as in “De Novo Assembly and Genome Annotation”. Snippy was then again used to call SNPs against this new “pangenomic reference”. Pseudo-whole genome alignments generated by snippy-core (v4.6.0) were then used as input to IQ-Tree(Minh et al., 2020) to estimate phylogenies for each ST. The best-fitting model of nucleotide substitution was identified using the IQ-Tree ModelFinder(Kalyaanamoorthy et al., 2017) (option *-m MFP*) and a maximum likelihood phylogeny generated with 10000 UltraFast(Hoang et al., 2018) bootstrap replicates (option *-bb 10000*). Consensus phylogenies were then used as input to ClonalFrameML(Didelot and Wilson, 2015) (v1.12) to identify putative recombinant regions, which were subsequently masked using the masrc-svg(Kwong and Seemann, n.d.) (v0.5) python script. Masked alignments were once again used as input to IQ-Tree, which was run as above to produce final phylogenies. Snp-dists(Seemann, n.d.) (v0.7.0) was used to generate SNP distance matrices for each ST.

SNP calling was also performed for all STs together against a single reference (*S. maltophilia* strain K279a, NCBI RefSeq Accession GCF_000072485.1) using Snippy as above. Snp-dists was used to obtain pairwise SNP distances between all pairs of isolates.

### Analysis of Possible Hypermutating Isolates

VCFTools(Danecek et al., 2011) (v0.1.16) was used to calculate transition/transversion ratios for all isolates using SNPs called against the *S. maltophilia* K279a reference. Bedtools(Quinlan and Hall, 2010) (v2.30.0) was used to extract mutations in mismatch repair genes (*mutS, mutL*, and *uvrD*).

### Multi-mutated Gene Analysis

For each ST, we identified all *S. maltophilia* genes with mutations that accumulated within a patient (for each patient) over the course of their infection (termed SmCF genes). That is, genes with mutations that were present in ≥1 isolates relative to their other isolates (of the same ST) of a given patient. All genes with such mutations were included, regardless of whether other patients did/did not have mutations in the same genes. We did not differentiate the “direction” of mutation and try to determine which allele at a given locus was ancestral vs. acquired but rather simply noted which genes had any mutations in them. This set of genes/mutations was therefore considered to include all mutations arising during infection in the CF lung (due to genetic drift and/or selective pressures). In contrast, genes with mutations segregating between isolates from different patients but not within any patient’s isolates were taken to represent mutations that arose prior to infection in CF and/or mutations defining separate strains of *S. maltophilia*, and not adaptation to the CF lung environment (termed non-adaptive genes/mutations). We note that it is possible that what we identified as non-adaptive mutations may have arisen and quickly fixed during infection in the CF lung if the earliest sequenced isolates did not include the hypothetical “first” infecting isolates, and so may have been incorrectly assigned as having occurred outside CF lung infection. Accordingly, genes denoted as SmCF therefore represent a conservative minimal set and were defined to minimize the type I error rate (false positives) at the cost of an increased type II error rate (false negatives).

Each SmCF/non-adaptive gene was then classified as multi-mutated if it had ≥2 mutations at different positions within the gene or different mutations at the same position, regardless of whether these occurred within one/multiple patients/isolates. Multi-mutated genes were further subdivided into *i)* multi-mutated across STs, if the contributing mutations occurred in ≥2 STs, or *ii)* multi-mutated within STs, if the multiple mutations were limited to a single ST. Genes that were both multi-mutated across and within STs were classified as multi-mutated across STs for purposes of statistical analysis. Only synonymous, non-synonymous, and stop gained mutations were considered for statistical testing. Other types of mutations recovered but not analyzed included intergenic mutations, start gained mutations, stop lost mutations, splice variants, and combinations of these mutation types as annotated by SnpEff. CF mutations were acquired from all STs with ≥1 patients with ≥2 isolates; this included all STs except ST-39. Non-adaptive mutations were acquired from all multi-patient STs; this included STs 5, 199, 220, and 224. Comparisons of the distributions of synonymous, non-synonymous, and stop mutations between multi-mutated and non-multi-mutated genes was performed using Fisher’s exact tests in GraphPad Prism (v9.4.1).

# Supplementary Figures and Tables

Please note that all Supplementary Figures are also available from a figshare digital repository: <https://doi.org/10.6084/m9.figshare.c.6465634.v1>.

## Supplementary Figures


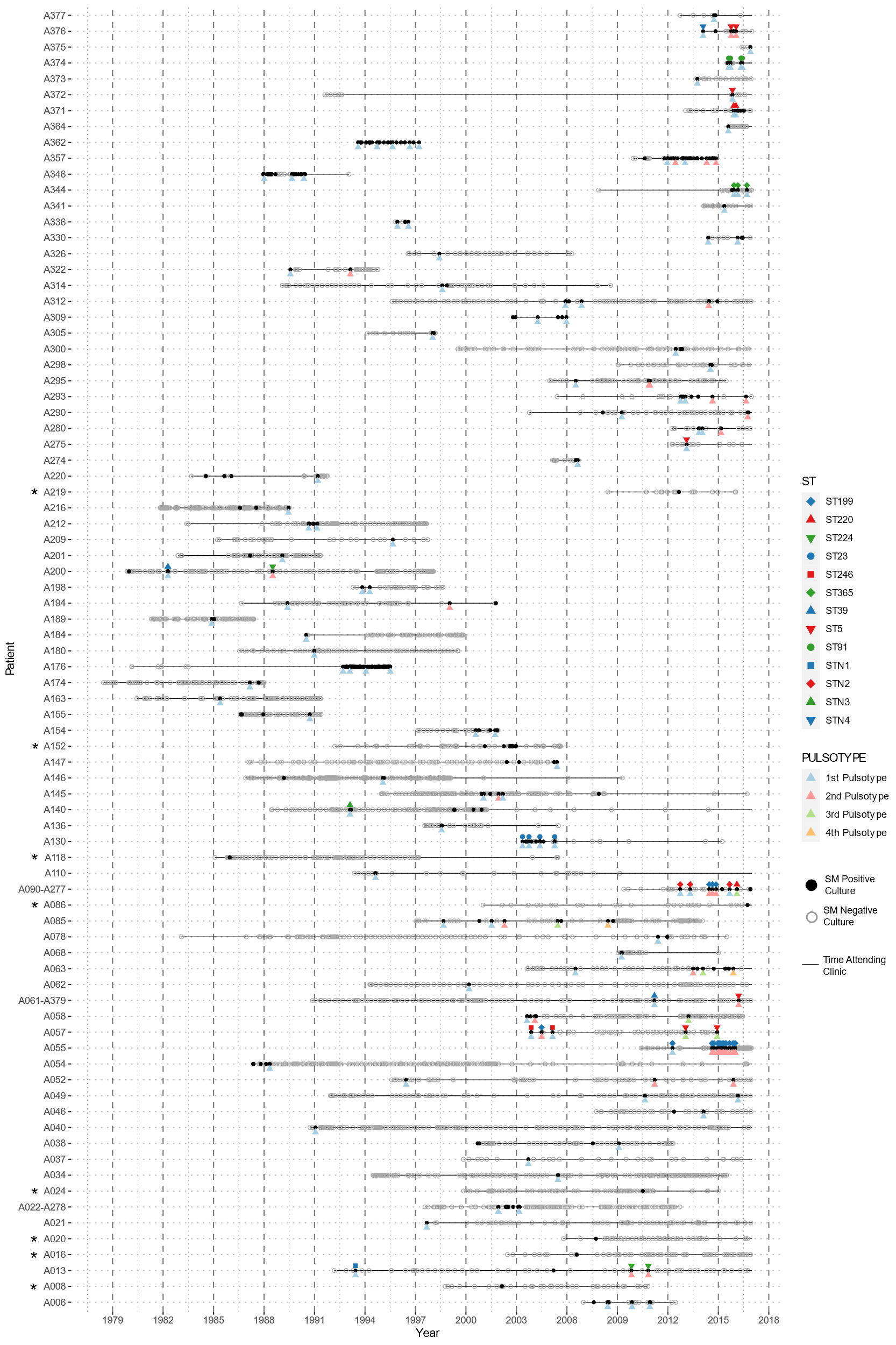


Supplementary Figure 1. Culture positivity timeline (x-axis) of pwCF (y-axis) with at least one S. maltophilia positive sputum culture. For each pwCF, the black line represents the time the patient attended the Southern Alberta Adult CF Clinic. Empty grey circles represent S. maltophilia negative sputum cultures, while filled black circles represent positive cultures. The row of colored triangles immediately underneath the black line represents isolates typed by PFGE and whether the recovered pulsotype was the first (blue), second (red), third (green), or fourth (yellow) distinct pulsotype recovered in each individual patient. These colors have no meaning between pwCF. The row of colored shapes immediately above the black line represents isolates typed by WGS and their MLST sequence types. Isolates with the same sequence types will have the same color/shape combination, and this is consistent between pwCF. PwCF marked with an asterisk (*) are those for whom no cultures were typed by PFGE (or WGS).


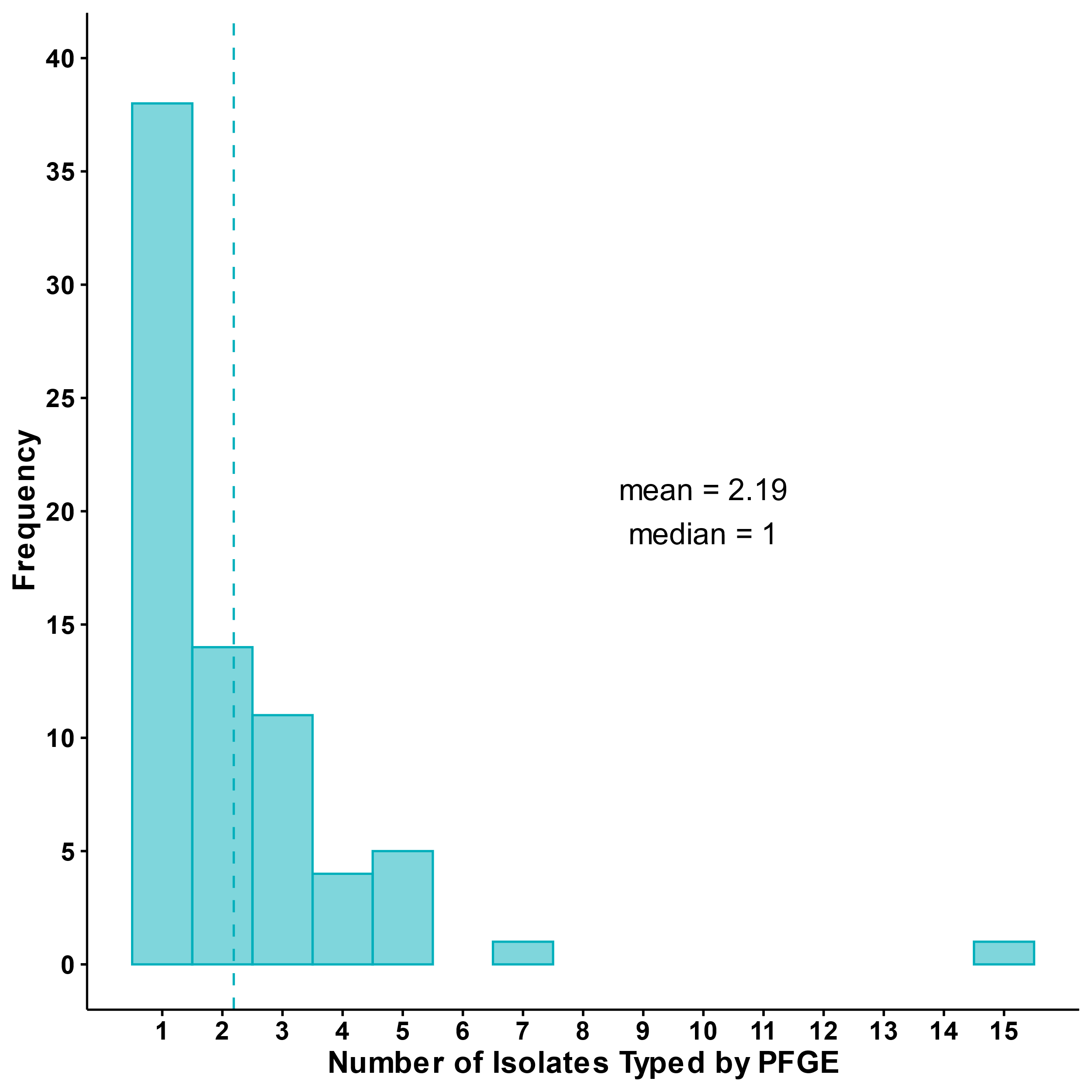


**Supplementary Figure 2. Distribution of the number of isolates typed by PFGE per pwCF.**


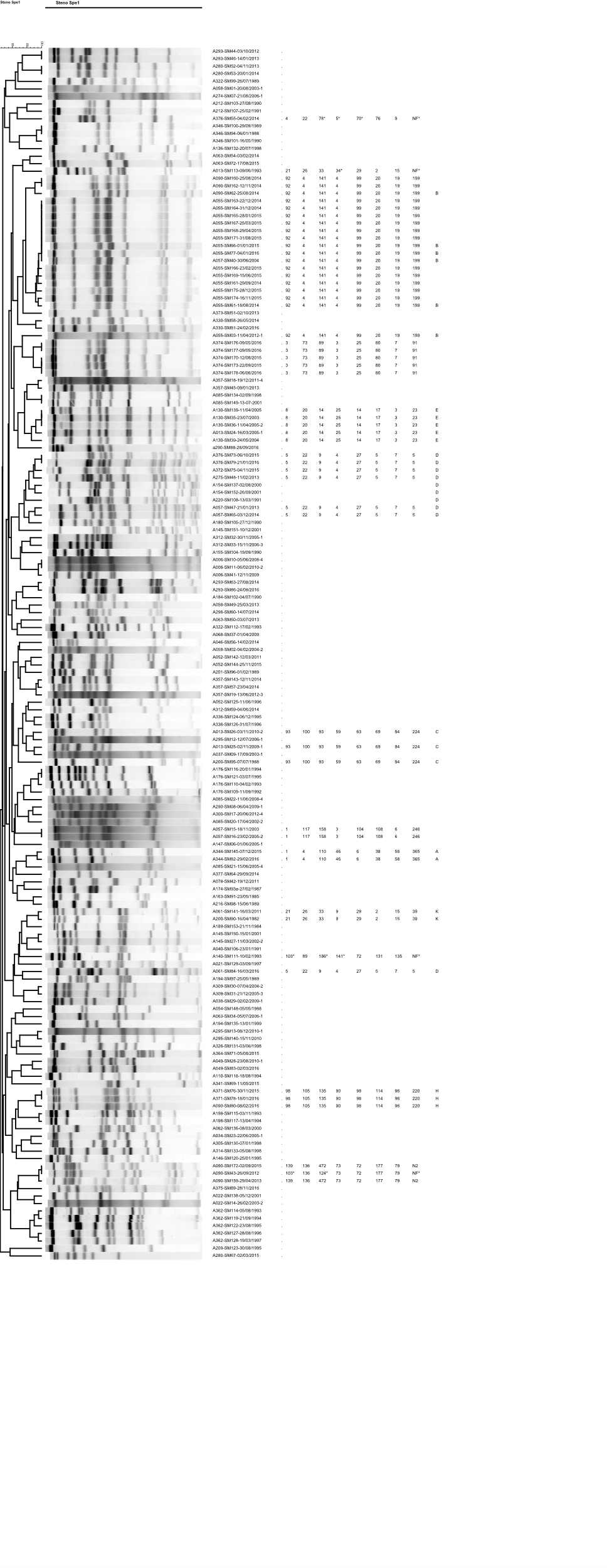


**Supplementary Figure 3. Pulsed-field gel electrophoresis dendrogram showing relationships among all 162 *S. maltophilia* isolates typed by pulsed-field gel electrophoresis.** Dendrogram was constructed with 1.5% optimization and 2% tolerance.


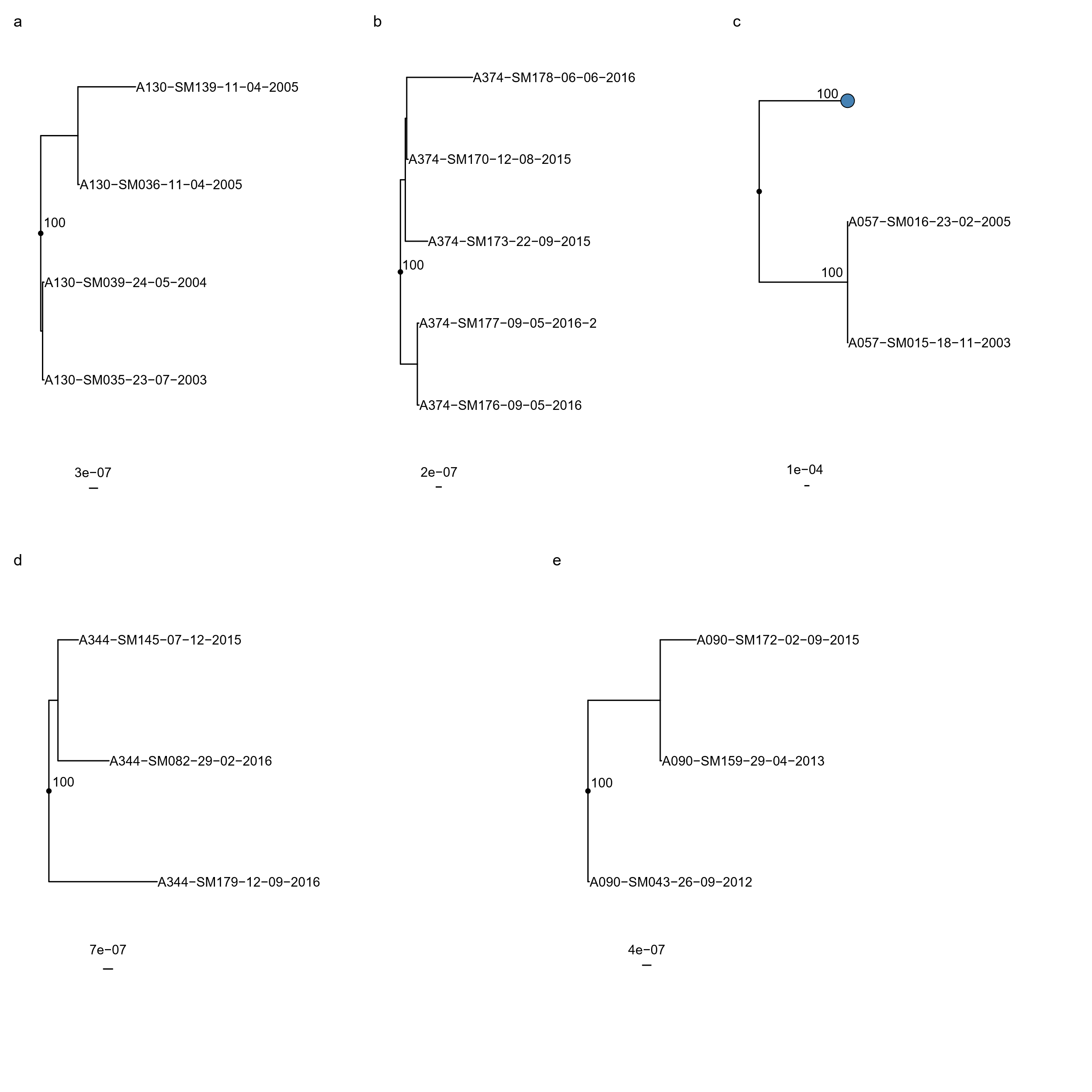


Supplementary Figure 4. Recombination-corrected maximum likelihood phylogenies of isolates belonging to non-shared STs: (A) ST-23, (B) ST-91, (C) ST-246, (D) ST-365, (E) ST-Novel 2. Each phylogeny is rooted at the midpoint of the branch where outgroups attach. In (C), the outgroups are represented by the blue circle. UltraFast bootstrap support is indicated only in clades with ≥95% support. Scale bars are in units of SNPs/site. Isolate names are presented in the format “Patient_Identification_Number-Isolate_Identification_Number-dd-mm-yyyy”.


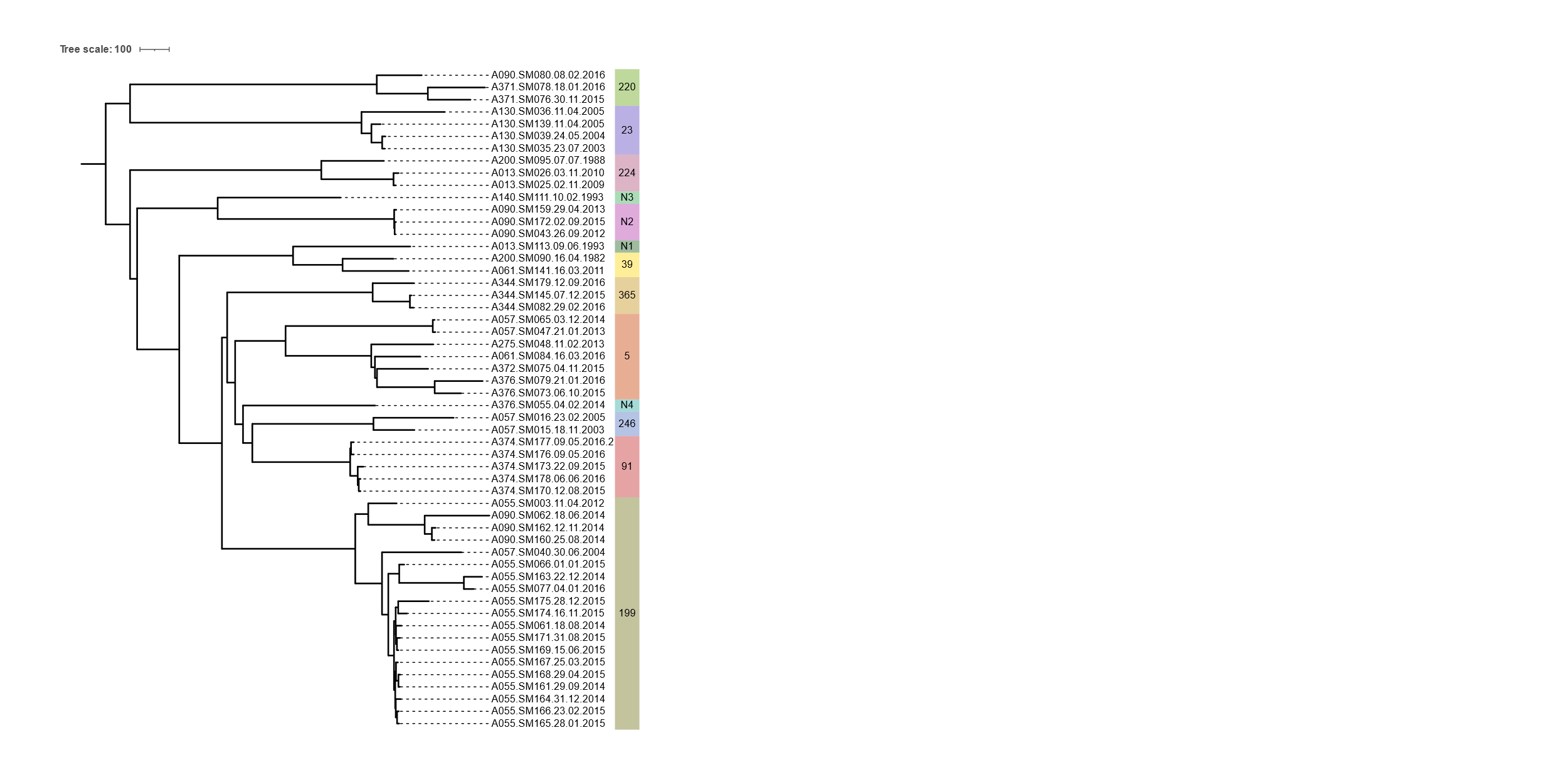


Supplementary Figure 5. Neighbor-joining phylogeny constructed from gene presence/absence data among all isolates sequenced in this study. STs are indicated by text and colored bands on the right. Isolate names are presented in the format “Patient_Identification_Number-Isolate_Identification_Number-dd-mm-yyyy”.

# References

Bolger, A. M., Lohse, M., and Usadel, B. (2014). Trimmomatic: a flexible trimmer for Illumina sequence data. *Bioinformatics* 30, 2114–2120. doi: 10.1093/bioinformatics/btu170.

Camacho, C., Coulouris, G., Avagyan, V., Ma, N., Papadopoulos, J., Bealer, K., et al. (2009). BLAST+: architecture and applications. *BMC Bioinformatics* 10, 421. doi: 10.1186/1471-2105-10-421.

Chauhan, A., Green, S., Pathak, A., Thomas, J., and Venkatramanan, R. (2013). Whole-genome sequences of five oyster-associated bacteria show potential for crude oil hydrocarbon degradation. *Genome Announc* 1, e00802-13. doi: 10.1128/genomeA.00802-13.

Danecek, P., Auton, A., Abecasis, G., Albers, C. A., Banks, E., DePristo, M. A., et al. (2011). The variant call format and VCFtools. *Bioinformatics* 27, 2156–2158. doi: 10.1093/bioinformatics/btr330.

Davis, J. J., Wattam, A. R., Aziz, R. K., Brettin, T., Butler, R., Butler, R. M., et al. (2020). The PATRIC Bioinformatics Resource Center: expanding data and analysis capabilities. *Nucleic Acids Research* 48, D606–D612. doi: 10.1093/nar/gkz943.

Didelot, X., and Wilson, D. J. (2015). ClonalFrameML: Efficient Inference of Recombination in Whole Bacterial Genomes. *PLoS Comput Biol* 11. doi: 10.1371/journal.pcbi.1004041.

enaBrowserTools (2022). Available at: https://github.com/enasequence/enaBrowserTools.

Esposito, A., Pompilio, A., Bettua, C., Crocetta, V., Giacobazzi, E., Fiscarelli, E., et al. (2017). Evolution of Stenotrophomonas maltophilia in Cystic Fibrosis Lung over Chronic Infection: A Genomic and Phenotypic Population Study. *Front Microbiol* 8. doi: 10.3389/fmicb.2017.01590.

FastQC (n.d.). Available at: https://www.bioinformatics.babraham.ac.uk/projects/fastqc/.

Gröschel, M. I., Meehan, C. J., Barilar, I., Diricks, M., Gonzaga, A., Steglich, M., et al. (2020). The phylogenetic landscape and nosocomial spread of the multidrug-resistant opportunist Stenotrophomonas maltophilia. *Nat Commun* 11, 2044. doi: 10.1038/s41467-020-15123-0.

Gupta, A., Jordan, I. K., and Rishishwar, L. (2017). stringMLST: a fast k-mer based tool for multilocus sequence typing. *Bioinformatics* 33, 119–121. doi: 10.1093/bioinformatics/btw586.

Hoang, D. T., Chernomor, O., von Haeseler, A., Minh, B. Q., and Vinh, L. S. (2018). UFBoot2: Improving the Ultrafast Bootstrap Approximation. *Mol Biol Evol* 35, 518–522. doi: 10.1093/molbev/msx281.

Kalyaanamoorthy, S., Minh, B. Q., Wong, T. K., von Haeseler, A., and Jermiin, L. S. (2017). ModelFinder: Fast Model Selection for Accurate Phylogenetic Estimates. *Nat Methods* 14, 587–589. doi: 10.1038/nmeth.4285.

Kanamori, H., Parobek, C. M., Weber, D. J., van Duin, D., Rutala, W. A., Cairns, B. A., et al. (2016). Next-Generation Sequencing and Comparative Analysis of Sequential Outbreaks Caused by Multidrug-Resistant Acinetobacter baumannii at a Large Academic Burn Center. *Antimicrob Agents Chemother* 60, 1249–1257. doi: 10.1128/AAC.02014-15.

Kwong, J., and Seemann, T. (n.d.). maskrc-svg. Available at: https://github.com/kwongj/maskrc-svg.

Letunic, I., and Bork, P. (2021). Interactive Tree Of Life (iTOL) v5: an online tool for phylogenetic tree display and annotation. *Nucleic Acids Research* 49, W293–W296. doi: 10.1093/nar/gkab301.

Lira, F., Berg, G., and Martínez, J. L. (2017). Double-Face Meets the Bacterial World: The Opportunistic Pathogen Stenotrophomonas maltophilia. *Front Microbiol* 8, 2190. doi: 10.3389/fmicb.2017.02190.

Minh, B. Q., Schmidt, H. A., Chernomor, O., Schrempf, D., Woodhams, M. D., von Haeseler, A., et al. (2020). IQ-TREE 2: New Models and Efficient Methods for Phylogenetic Inference in the Genomic Era. *Molecular Biology and Evolution* 37, 1530–1534. doi: 10.1093/molbev/msaa015.

Niu, B., Paulson, J. N., Zheng, X., and Kolter, R. (2017). Simplified and representative bacterial community of maize roots. *Proc Natl Acad Sci U S A* 114, E2450–E2459. doi: 10.1073/pnas.1616148114.

Ochoa-Sánchez, L. E., and Vinuesa, P. (2017). Evolutionary Genetic Analysis Uncovers Multiple Species with Distinct Habitat Preferences and Antibiotic Resistance Phenotypes in the Stenotrophomonas maltophilia Complex. *Front Microbiol* 8, 1548. doi: 10.3389/fmicb.2017.01548.

Pak, T. R., Altman, D. R., Attie, O., Sebra, R., Hamula, C. L., Lewis, M., et al. (2015). Whole-genome sequencing identifies emergence of a quinolone resistance mutation in a case of Stenotrophomonas maltophilia bacteremia. *Antimicrob Agents Chemother* 59, 7117–7120. doi: 10.1128/AAC.01723-15.

Parkins, M. D., Glezerson, B. A., Sibley, C. D., Sibley, K. A., Duong, J., Purighalla, S., et al. (2014). Twenty-Five-Year Outbreak of Pseudomonas aeruginosa Infecting Individuals with Cystic Fibrosis: Identification of the Prairie Epidemic Strain. *J. Clin. Microbiol.* 52, 1127–1135. doi: 10.1128/JCM.03218-13.

Parks, D. H., Rinke, C., Chuvochina, M., Chaumeil, P.-A., Woodcroft, B. J., Evans, P. N., et al. (2017). Recovery of nearly 8,000 metagenome-assembled genomes substantially expands the tree of life. *Nat Microbiol* 2, 1533–1542. doi: 10.1038/s41564-017-0012-7.

Patil, P. P., Midha, S., Kumar, S., and Patil, P. B. (2016). Genome Sequence of Type Strains of Genus Stenotrophomonas. *Front Microbiol* 7, 309. doi: 10.3389/fmicb.2016.00309.

Quinlan, A. R., and Hall, I. M. (2010). BEDTools: a flexible suite of utilities for comparing genomic features. *Bioinformatics* 26, 841–842. doi: 10.1093/bioinformatics/btq033.

Roach, D. J., Burton, J. N., Lee, C., Stackhouse, B., Butler-Wu, S. M., Cookson, B. T., et al. (2015). A Year of Infection in the Intensive Care Unit: Prospective Whole Genome Sequencing of Bacterial Clinical Isolates Reveals Cryptic Transmissions and Novel Microbiota. *PLoS Genet* 11, e1005413. doi: 10.1371/journal.pgen.1005413.

Seemann, T. (n.d.). Snippy. Available at: https://github.com/tseemann/snippy.

Seemann, T. (n.d.). snp-dists. Available at: https://github.com/tseemann/snp-dists.

Silva, M., Machado, M. P., Silva, D. N., Rossi, M., Moran-Gilad, J., Santos, S., et al. (2018). chewBBACA: A complete suite for gene-by-gene schema creation and strain identification. *Microb Genom* 4, e000166. doi: 10.1099/mgen.0.000166.

Tonkin-Hill, G., MacAlasdair, N., Ruis, C., Weimann, A., Horesh, G., Lees, J. A., et al. (2020). Producing polished prokaryotic pangenomes with the Panaroo pipeline. *Genome Biology* 21, 180. doi: 10.1186/s13059-020-02090-4.

Wick, R. R., Judd, L. M., Gorrie, C. L., and Holt, K. E. (2017). Unicycler: Resolving bacterial genome assemblies from short and long sequencing reads. *PLOS Computational Biology* 13, e1005595. doi: 10.1371/journal.pcbi.1005595.

Yero, D., Huedo, P., Conchillo-Solé, O., Martínez-Servat, S., Mamat, U., Coves, X., et al. (2020). Genetic Variants of the DSF Quorum Sensing System in Stenotrophomonas maltophilia Influence Virulence and Resistance Phenotypes Among Genotypically Diverse Clinical Isolates. *Front Microbiol* 11, 1160. doi: 10.3389/fmicb.2020.01160.

Zhou, Z., Alikhan, N.-F., Sergeant, M. J., Luhmann, N., Vaz, C., Francisco, A. P., et al. (2018). GrapeTree: visualization of core genomic relationships among 100,000 bacterial pathogens. *Genome Res* 28, 1395–1404. doi: 10.1101/gr.232397.117.
